# Positive and negative AIT trials: What makes the difference?

**DOI:** 10.1007/s40629-018-0058-y

**Published:** 2018-05-24

**Authors:** Roy Gerth van Wijk

**Affiliations:** Section of Allergology, Department of Internal Medicine, Erasmus Medical Centre, ’s Gravendijkwal 230, 3015 CE Rotterdam, The Netherlands

**Keywords:** Allergen immunotherapy, Randomised clinical trial, Endpoints, Negative study, Phase II trial, Phase III trial

## Abstract

**Background:**

Allergen immunotherapy has proven to be efficacious in allergic rhinitis and asthma. However, results from randomised clinical trials may vary substantially. Clinical trials may unexpectedly fail. The purpose of this review is to discuss the possible factors that may contribute to a successful or unsuccessful study.

**Methods:**

Descriptive review exploring the possible causes of negative outcomes in allergen immunotherapy trials.

**Results:**

A series of factors may lead to negative results. Among of these are underpowering of the study, low allergen content in tested extracts, insufficient allergen exposure during monitoring and recruitment of inappropriate patients. In addition, the choice of the primary endpoint may be critical.

**Discussion:**

A clinical trial aims to evaluate the efficacy of an agent. However, studies with potential effective compounds may fail because of methodical issues. Sometimes, they are the cause of discrepancies between successful phase II and unsuccessful phase III trials. To understand more about failure of studies, investigators and editors should be encouraged to publish negative trials.

## Introduction

Allergen immunotherapy (AIT) already exists for more than a century. Leonard Noon described the first inoculation with grass pollen extract demonstrating that repeated subcutaneous injection leads to diminished conjunctival sensitivity [1]. The first randomised controlled trial (RCT) of subcutaneous grass pollen immunotherapy was performed by William Frankland [2]. He successfully demonstrated the superiority of grass pollen extract over placebo. In later years much attention has been paid to alternative routes of administration. In 1986 the first double blind, placebo-controlled sublingual AIT in patients with allergic rhinitis due to house dust mite demonstrated diminished nasal responsiveness to house dust mites following active treatment [3].

Although subcutaneous and sublingual allergen immunotherapy for both rhinitis [4] and asthma [5] have proven efficacy as demonstrated in recent large systematic reviews, a wide variation in study results can be seen. Meta-analyses from AIT studies are also characterised by considerable heterogeneity [6, 7].

AIT may simply not work because the newly tested compound is not capable to induce a therapeutic effect. An effect can be unexpected. For instance, intradermal grass pollen extract immunotherapy led to the suppression of late phase skin reactions, whereas there was no difference in primary endpoint (symptom-medication score) between active and placebo treatment [8]. Paradoxically, some secondary endpoints were worse in the intradermal immunotherapy group (nasal symptoms, visual analogue scale, asthma symptoms and symptom free days).

Although the outcome of a randomised clinical trial will be determined by the efficacy of the active compound, other factors may influence the result of an AIT trial. The purpose of this paper is to discuss the possible factors that may contribute to a successful or unsuccessful study (Table 1).

**Table 1:** Reasons for an unsuccessful allergen immunotherapy (AIT) study

The study is underpowered
The tested agent does not contain sufficient allergen or the agent has been administered too infrequently
Participants are not sufficiently exposed to the culprit allergen during the assessment phase
There are flaws in the recruitment of participants, for instance too many patients with mild disease are included
The primary endpoint is not met (from a regulator’s point of view) and/or the primary endpoint is not appropriate for the study

## Possible factors that may influence the outcome an AIT trial

### Underpowering

A study should be well designed and well powered. Bousquet et al. analysed 46 subcutaneous (SCIT) and 48 sublingual (SLIT) double blind, placebo-controlled randomised immunotherapy trials [9] using the Consolidated Standards of Reporting Trials (CONSORT) checklist. These trials were published between 1996 and 2009. Important flaws in the reports were the absence of a flowchart and description of dropouts, inadequate or incomplete randomisation, absence of a power-analysis, absence of an intention-to-treat (ITT) analysis or the use of a modified ITT analysis. Most striking was that in 33 and 27% respectively of the SCIT and SLIT studies a power analysis was not reported.

Underpowering has been put forward as one of the explanations for a negative sublingual AIT study among asthmatic children [10]. Particularly, when two active treatments are compared a power calculation is crucial. In a placebo-controlled, double-dummy study both birch pollen SCIT and SLIT appeared superior to placebo; however a significant difference between both treatments could not be found [11]. The authors indeed state that to demonstrate a difference larger groups than the 19 SCIT and 15 SLIT patients should be included.

### Allergen content of the tested agent

An important reason for ineffective treatment may be the lack of sufficient allergen content. When comparing extracts from different manufacturers a wide variation in allergen content can be found. In a study on allergen content of grass pollen preparations for sublingual immunotherapy the differences between the immunotherapeutics with the lowest vs highest maintenance dose amounted to a factor of 31 for protein, 26–60 for allergen, depending on the used serum pool, and up to 108 for the daily Phl p 5 dosages [12]. Such differences may easily explain the heterogeneity in study results often seen in AIT trials.

Also extracts containing sufficient amounts of allergen may be unsuccessful if the frequency of administration is too low to demonstrate a substantial clinical effect. Examples are a 2-year grass pollen tablet study with only clinical efficacy in the 2^nd^ year using the grass pollen tablets 3 times per week [13] and a negative grass pollen drops study among children and adolescents, administering 2 doses per week [14].

### Allergen exposure

It is clear that without sufficient natural allergen exposure a study on the efficacy of AIT is doomed to fail. A well-designed phase II/III randomised clinical trial (RCT) evaluating 2 maintenance doses of immunologically enhanced standardised quality grass pollen immunotherapy failed to demonstrate efficacy compared with placebo in spite of dose-dependent increases in IgG_4_ and blocking antibodies, which were comparable with previous grass SCIT and grass SLIT tablet trials [15]. The inconclusive results were most probably influenced by a very low grass pollen season with a mean grass pollen count of 27.0 grains/m^3^.

A post hoc analysis comprising the data from 2363 patients participating in six grass pollen–tablet studies and seven grass pollen seasons across North America and Europe looked at the association between the magnitude of efficacy measurements and pollen exposure [16]. It appeared that the magnitude of effect was highly dependent on pollen exposure. The relative difference between placebo and active treatment expressed in total combined symptom score (TCS) could be predicted from the formula: TCS = 12% = 0.35% × pollen count (*p* = 0.003; R^2^ = 0.66).

It is obvious that pollen AIT should be monitored during the pollen season. It is less clear whether peak exposure to house dust mites should be taken into account when designing a trial. Although fluctuation in house dust mite (HDM) exposure is less pronounced in Dutch houses higher levels of Der p1 have been seen August–October compared to January–May. These high levels were accompanied by decrease in bronchial hyperreactivity [17]. However, very few HDM studies measured allergen exposure at baseline [18], whereas one study only determined Der p1 before and during the study.

### Characteristics of the patient

For inclusion in AIT trials patients need to be sensitised to the allergen that will be evaluated, moreover a history is required that matches with the sensitisation. For a successful trial it is obvious that room for improvement is needed. Patients with mild disease may not further improve on active treatment, moreover long-lasting immunotherapy is also not intended for such patients. There are several ways to insure that moderate to severe rhinitis patients are being recruited. Several investigators recruited patients with poor symptom control in spite of the use of adequate pharmacotherapy [19–23]. Alternatively, a retrospective symptom score can be used [14, 24]. The disadvantage of the latter approach is that these scores can be hampered by recall bias. An observational period or baseline assessment is also possible [25, 26] which will lengthen the study. The importance of disease severity is underlined by a study from Howarth evaluating 3 multicentre trials [27]. Based on symptom severity patients were allocated to low, medium and high tertiles. The treatment effect increased with greater disease severity.

### The choice of the primary endpoint

A RCT has a primary endpoint as a measure of success of the trial. The study is ideally powered for the primary endpoint. Separately, other endpoints of interest are put forward as secondary endpoint. The variety of possible endpoints in AIT trials with rhinoconjunctivitis patients has led to a position paper on this topic [28]. Although not validated, a combined symptom medication score is currently proposed as the standard endpoint for AIT studies in rhinitis patients. The absence of clearly defined endpoints will hamper progress in this field. A negative Cochrane meta-analysis on sublingual immunotherapy in asthma concluded that lack of data for important outcomes such as exacerbations and quality of life and use of different unvalidated symptom and medication score have limited the ability to draw a clinically useful conclusion [29]. Recently, a successful study with HDM tablets in partly to uncontrolled asthmatics has been published [30]. Active treatment reduced the time to develop an asthma exacerbation, the primary outcome. Asthma control and quality of life scores, secondary outcomes, were not affected. These outcome measures were probably not suitable for the design of the study as patients were tapering down their steroids until the first asthma exacerbation. It is conceivable that in this short period quality of life or asthma control would not have been substantially affected. If one of these outcome measures were chosen as primary outcome, the study would have failed.

Other examples of the importance of outcome measures are the Preventive Allergy Treatment (PAT; [31]) and Grass SLIT tablet Asthma Prevention (GAP; [32]) study. The PAT study is a RCT to the effect of subcutaneous grass pollen immunotherapy on the development of asthma [31]. The hypothesis was that immunotherapy may prevent asthma. And indeed, in this trial comprising 203 children among those without asthma actively treated children had significantly fewer asthma symptoms (odds ratio [OR] 2.52; *p* < 0.05) after 3 years. The effect remained up to 10 years after starting treatment [33]. In contrast to the open and controlled but not placebo-controlled PAT study, the GAP trial, aiming to evaluate the preventive effect of SLIT, was placebo controlled with 3 years treatment and 2 years blinded follow-up [32]. A total of 812 children were included and again in the actively treated group the risk of experiencing asthma symptoms or using asthma medication (OR 0.66; *p* < 0.036) was lower. However, in the latter study there was no difference in time to onset of asthma, the primary outcome of the GAP study. For the diagnosis of asthma documentation of a reversible lung function impairment was required. Children with frequent episodes of asthma symptoms but a normal lung function at the time of the trial visits were not recorded as having asthma. By choosing another endpoint the GAP study was—from a regulator’s perspective—not successful, whereas the results were at least promising.

Some studies that did not reach the primary endpoint are still considered successful or at least promising. In 2006 a small phase 2 trial of ragweed pollen conjugated to a phosphorothioate oligodeoxyribonucleotide immunostimulatory sequence of DNA containing a CpG motif was reported [34]. Six pre-seasonal injections only appeared to be clinically effective during 2 ragweed seasons. The primary endpoint—vascular leakage and inflammation during nasal allergen challenge—however was not reached.

A recent study looking at the effect of recombinant fusion proteins consisting of non-allergenic grass pollen peptides and the hepatitis B preS protein reported that active treatment failed to meet the primary endpoint (symptom-medication score). However a post hoc analysis with patients having specific IgE ≥3.5 kUl/l demonstrated statistically significant improvement in symptom-medication scores in the 2^nd^ year of treatment. In addition, visual analogue scores and quality of life improved significantly [35].

A successful phase II peanut immunotherapy study has been published by Sampson et al. [36]. Using the technique of epicutaneous immunotherapy with peanut, they demonstrated in a one year study that active treatment generate a response rate to treatment of 25% (95% confidence interval [CI] 7.7–42.3%, *p* = 0.01) over placebo. The difference was even higher in children (34.2%; 95% CI 11.1–57.3%, *p* = 0.08). Successful treatment was achieved when an eliciting dose was reached ≥10 times increase and/or ≥1000 mg of peanut protein. In a recent phase 3 study the difference in response rate over placebo was lower, but still significant (21.7%, 95% CI 12.4–29.8%; [37]). However, in the statistical analysis plan submitted to the FDA it was agreed to the lower bound of the CI should be 15%. From that perspective, the aim of this study was not reached.

One might say that success or failure of a trial is also a matter of definition.

### The placebo effect

AIT are characterised by a substantial placebo effect. In a survey from 2013 it was shown that the mean placebo effect in 5 SCIT trials amounts to 41% [38]. This was not seen with SLIT, but in this analysis only 1 SLIT study was included. Two recent unpublished AIT trials were hampered by substantial placebo effects. In a field study with cat-peptides the active treatment group showed a reduction of 61% in symptoms with a comparable reduction of 59.5% in the placebo group [39]. The results were remarkable as a phase II study evaluating efficacy by use of allergen exposure chambers was successful [40]. Also a field study with HDM peptides was hampered by a placebo effect of around 40%, comparable with the active treatment effects of three different doses [41].

Large placebo effects are more rule than exception also in successful AIT studies; thus it is difficult to state that only the placebo effect is responsible for the negative outcome of a study.

### The difference between a phase II and III study

The above mentioned studies demonstrate that the results of phase II and III studies can differ. The phase II and III studies may differ in design and are therefore not fully comparable. In the cat studies not only cat exposure differed (allergen exposure chamber versus natural exposure), but also the study population differed with patients without cats in the phase II study and cat-owners in the phase III study. It is a matter of debate whether the cat owners were less likely to respond to treatment than those who were not continuously exposed to cat allergen.

The study from Creticos et al. [34] which tested a ragweed vaccine conjugated with a toll-like receptor 9 agonist resulted in a large phase III study with 30 centres and 738 subjects. Unfortunately this study failed to demonstrate any efficacy of the ragweed vaccine [42]. It has been stated that the 3 patients groups (2 doses, 1 placebo) had no measurable disease during the ragweed season. This suggests that either there was no exposure to ragweed pollen or the patients were misclassified not having sufficient ragweed pollen allergy to establish a therapeutic response.

## Conclusion

There are many reasons for failure of a RCT evaluating the efficacy of AIT. Of course, the main reason for failure is that the agent to be tested does not have the biological activity to generate a therapeutic effect. Modifications and new ways of administration may be the cause. An allergenic extract may contain an insufficient amount of allergen. However, other factors that are not related to the real efficacy of the active compound may determine the outcome of trials. The design of the study, insufficient allergen exposure during monitoring, inclusion of inappropriate patients and choosing the wrong endpoints may lead to a negative study result that does not represent the real efficacy of test agent. Particularly, when a successful phase II trial is followed by failure of phase III studies, factors that may negatively influence the outcome of the latter studies have to be analysed. An important barrier for understanding the reasons for unsuccessful phase III trials and negative trials in general is the unavailability of published data. Investigators should be encouraged to publish their negative trials and editors should consider the value of such reports in their journal.

## Abbreviations

AIT: Allergen immunotherapy
CI: Confidence interval
CONSORT: Consolidated Standards of Reporting Trials
GAP: Grass SLIT tablet Asthma Prevention
HDM: House dust mite
ITT: Intention-to-treat
OR: Odds ratio
PAT: Preventive Allergy Treatment
RCT: Randomised clinical trial
RCT: Randomised controlled trial
SCIT: Subcutaneous immunotherapy
SLIT: Sublingual immunotherapy
TCS: Total combined symptom score

## References

[1] Noon LA (1911). Prophylactic inoculation against hay fever. Lancet.

[2] Frankland AW, Augustin R (1954). Prophylaxis of summer hay-fever and asthma: a controlled trial comparing crude grass-pollen extracts with the isolated main protein component. Lancet.

[3] Scadding GK, Brostoff J (1986). Low dose sublingual therapy in patients with allergic rhinitis due to house dust mite. Clin Allergy.

[4] Dhami S, Nurmatov U, Arasi S, Khan T, Asaria M, Zaman H (2017). Allergen immunotherapy for allergic rhinoconjunctivitis: a systematic review and meta-analysis. Allergy.

[5] Dhami S, Kakourou A, Asamoah F, Agache I, Lau S, Jutel M (2017). Allergen immunotherapy for allergic asthma: a systematic review and meta-analysis. Allergy.

[6] Calderon MA, Alves B, Jacobson M, Hurwitz B, Sheikh A, Durham S (2007). Allergen injection immunotherapy for seasonal allergic rhinitis. Cochrane Database Syst Rev.

[7] Radulovic S, Wilson D, Calderon M, Durham S (2011). Systematic reviews of sublingual immunotherapy (SLIT). Allergy.

[8] Slovick A, Douiri A, Muir R, Guerra A, Tsioulos K, Hay E (2017). Intradermal grass pollen immunotherapy increases TH2 and IgE responses and worsens respiratory allergic symptoms. J Allergy Clin Immunol.

[9] Bousquet PJ, Calderon MA, Demoly P, Larenas D, Passalacqua G, Bachert C (2011). The Consolidated Standards of Reporting Trials (CONSORT) Statement applied to allergen-specific immunotherapy with inhalant allergens: a Global Allergy and Asthma European Network (GA(2)LEN) article. J Allergy Clin Immunol.

[10] Pham-Thi N, Scheinmann P, Fadel R, Combebias A, Andre C (2007). Assessment of sublingual immunotherapy efficacy in children with house dust mite-induced allergic asthma optimally controlled by pharmacologic treatment and mite-avoidance measures. Pediatr Allergy Immunol.

[11] Khinchi MS, Poulsen LK, Carat F, Andre C, Hansen AB, Malling HJ (2004). Clinical efficacy of sublingual and subcutaneous birch pollen allergen-specific immunotherapy: a randomized, placebo-controlled, double-blind, double-dummy study. Allergy.

[12] Sander I, Fleischer C, Meurer U, Bruning T, Raulf-Heimsoth M (2009). Allergen content of grass pollen preparations for skin prick testing and sublingual immunotherapy. Allergy.

[13] Smith H, White P, Annila I, Poole J, Andre C, Frew A (2004). Randomized controlled trial of high-dose sublingual immunotherapy to treat seasonal allergic rhinitis. J Allergy Clin Immunol.

[14] Roder E, Berger MY, Hop WC, Bernsen RM, de Groot H, Gerth van Wijk R (2007). Sublingual immunotherapy with grass pollen is not effective in symptomatic youngsters in primary care. J Allergy Clin Immunol.

[15] Kleine-Tebbe J, Walmar M, Bitsch-Jensen K, Decot E, Pfaar O, de Rojas DH (2014). Negative clinical results from a randomised, double-blind, placebo-controlled trial evaluating the efficacy of two doses of immunologically enhanced, grass subcutaneous immunotherapy despite dose-dependent immunological response. Clin Drug Investig.

[16] Durham SR, Nelson HS, Nolte H, Bernstein DI, Creticos PS, Li Z (2014). Magnitude of efficacy measurements in grass allergy immunotherapy trials is highly dependent on pollen exposure. Allergy.

[17] van der Heide S, de Monchy JG, de Vries K, Bruggink TM, Kauffman HF (1994). Seasonal variation in airway hyperresponsiveness and natural exposure to house dust mite allergens in patients with asthma. J Allergy Clin Immunol.

[18] Calderon MA, Casale TB, Nelson HS, Demoly P (2013). An evidence-based analysis of house dust mite allergen immunotherapy: a call for more rigorous clinical studies. J Allergy Clin Immunol.

[19] Dahl R, Kapp A, Colombo G, de Monchy JG, Rak S, Emminger W (2006). Efficacy and safety of sublingual immunotherapy with grass allergen tablets for seasonal allergic rhinoconjunctivitis. J Allergy Clin Immunol.

[20] Durham SR, Walker SM, Varga EM, Jacobson MR, O’Brien F, Noble W (1999). Long-term clinical efficacy of grass-pollen immunotherapy. N Engl J Med.

[21] Frew AJ, Powell RJ, Corrigan CJ, Durham SR, Group UKIS (2006). Efficacy and safety of specific immunotherapy with SQ allergen extract in treatment-resistant seasonal allergic rhinoconjunctivitis. J Allergy Clin Immunol.

[22] Varney VA, Gaga M, Frew AJ, Aber VR, Kay AB, Durham SR (1991). Usefulness of immunotherapy in patients with severe summer hay fever uncontrolled by antiallergic drugs. BMJ.

[23] Varney VA, Tabbah K, Mavroleon G, Frew AJ (2003). Usefulness of specific immunotherapy in patients with severe perennial allergic rhinitis induced by house dust mite: a double-blind, randomized, placebo-controlled trial. Clin Exp Allergy.

[24] Didier A, Worm M, Horak F, Sussman G, de Beaumont O, Le Gall M (2011). Sustained 3‑year efficacy of pre- and coseasonal 5‑grass-pollen sublingual immunotherapy tablets in patients with grass pollen-induced rhinoconjunctivitis. J Allergy Clin Immunol.

[25] Blaiss M, Maloney J, Nolte H, Gawchik S, Yao R, Skoner DP (2011). Efficacy and safety of timothy grass allergy immunotherapy tablets in North American children and adolescents. J Allergy Clin Immunol.

[26] Wahn U, Klimek L, Ploszczuk A, Adelt T, Sandner B, Trebas-Pietras E (2012). High-dose sublingual immunotherapy with single-dose aqueous grass pollen extract in children is effective and safe: a double-blind, placebo-controlled study. J Allergy Clin Immunol.

[27] Howarth P, Malling HJ, Molimard M, Devillier P (2012). Analysis of allergen immunotherapy studies shows increased clinical efficacy in highly symptomatic patients. Allergy.

[28] Pfaar O, Demoly P, Gerth van Wijk R, Bonini S, Bousquet J, Canonica GW (2014). Recommendations for the standardization of clinical outcomes used in allergen immunotherapy trials for allergic rhinoconjunctivitis: an EAACI position paper. Allergy.

[29] Normansell R, Kew KM, Bridgman AL (2015). Sublingual immunotherapy for asthma. Cochrane Database Syst Rev.

[30] Virchow JC, Backer V, Kuna P, Prieto L, Nolte H, Villesen HH (2016). Efficacy of a house dust mite sublingual allergen immunotherapy tablet in adults with allergic asthma: a randomized clinical trial. JAMA.

[31] Moller C, Dreborg S, Ferdousi HA, Halken S, Host A, Jacobsen L (2002). Pollen immunotherapy reduces the development of asthma in children with seasonal rhinoconjunctivitis (the PAT-study). J Allergy Clin Immunol.

[32] Valovirta E, Petersen TH, Piotrowska T, Laursen MK, Andersen JS, Sorensen HF (2018). Results from the 5‑year SQ grass sublingual immunotherapy tablet asthma prevention (GAP) trial in children with grass pollen allergy. J Allergy Clin Immunol.

[33] Jacobsen L, Niggemann B, Dreborg S, Ferdousi HA, Halken S, Host A (2007). Specific immunotherapy has long-term preventive effect of seasonal and perennial asthma: 10-year follow-up on the PAT study. Allergy.

[34] Creticos PS, Schroeder JT, Hamilton RG, Balcer-Whaley SL, Khattignavong AP, Lindblad R (2006). Immunotherapy with a ragweed-toll-like receptor 9 agonist vaccine for allergic rhinitis. N Engl J Med.

[35] Niederberger V, Neubauer A, Gevaert P, Zidarn M, Worm M, Aberer W (2018). Safety and efficacy of immunotherapy with the recombinant B‑cell epitope-based grass pollen vaccine BM32. J Allergy Clin Immunol.

[36] Sampson HA, Shreffler WG, Yang WH, Sussman GL, Brown-Whitehorn TF, Nadeau KC (2017). Effect of varying doses of epicutaneous immunotherapy vs placebo on reaction to peanut protein exposure among patients with peanut sensitivity: a randomized clinical trial. JAMA.

[37] Technologies D (2017). Results of phase III clinicical trial in peanut allergic children four to 11 years of age.

[38] Narkus A, Lehnigk U, Haefner D, Klinger R, Pfaar O, Worm M (2013). The placebo effect in allergen-specific immunotherapy trials. Clin Transl Allergy.

[39] Circassia (2016). Cat allergy phase III study June 2016 final.

[40] Patel D, Couroux P, Hickey P, Salapatek AM, Laidler P, Larche M (2013). Fel d 1‑derived peptide antigen desensitization shows a persistent treatment effect 1 year after the start of dosing: a randomized, placebo-controlled study. J Allergy Clin Immunol.

[41] Circassia (2017). Circassia announces top-line results from house dust mite allergy field study.

[42] Dynavax (2007). Dynavax reports interim TOLAMBA TM ragweed allergy results from DARTT trial 2007.

